# Cyclin-Dependent Kinase Inhibitor P1446A Induces Apoptosis in a JNK/p38 MAPK-Dependent Manner in Chronic Lymphocytic Leukemia B-Cells

**DOI:** 10.1371/journal.pone.0143685

**Published:** 2015-11-25

**Authors:** Cody Paiva, J. Claire Godbersen, Ryan S. Soderquist, Taylor Rowland, Sumner Kilmarx, Stephen E. Spurgeon, Jennifer R. Brown, Sreesha P. Srinivasa, Alexey V. Danilov

**Affiliations:** 1 Knight Cancer Institute, Oregon Health and Science University, Portland, OR, United States of America; 2 Dartmouth College, Hanover, NH, United States of America; 3 Duke University, Durham, NC, United States of America; 4 Medical Oncology, Dana-Farber Cancer Institute, Boston, MA, United States of America; 5 Piramal Healthcare Ltd., Mumbai, India; Duke University Medical Center, UNITED STATES

## Abstract

CDK (cyclin-dependent kinase) inhibitors have shown remarkable activity in CLL, where its efficacy has been linked to inhibition of the transcriptional CDKs (7 and 9) and deregulation of RNA polymerase and short-lived pro-survival proteins such as MCL1. Furthermore, ER (endoplasmic reticulum) stress has been implicated in CDK inhibition in CLL. Here we conducted a pre-clinical study of a novel orally active kinase inhibitor P1446A in CLL B-cells. P1446A inhibited CDKs at nanomolar concentrations and induced rapid apoptosis of CLL cells *in vitro*, irrespective of chromosomal abnormalities or *IGHV* mutational status. Apoptosis preceded inactivation of RNA polymerase, and was accompanied by phosphorylation of stress kinases JNK (c-Jun N-terminal kinase) and p38 MAPK (mitogen-activated protein kinase). Pharmacologic inhibitors of JNK/p38 MAPK conferred protection from P1446A-mediated apoptosis. Treatment with P1446A led to a dramatic induction of NOXA in a JNK-dependent manner, and sensitized CLL cells to ABT-737, a BH3-mimetic. We observed concurrent activation of apoptosis stress-inducing kinase 1 (ASK1) and its interaction with inositol-requiring enzyme 1 (IRE1) and tumor necrosis factor receptor-associated factor 2 (TRAF2) in CLL cells treated with P1446A, providing insights into upstream regulation of JNK in this setting. Consistent with previous reports on limited functionality of ER stress mechanism in CLL cells, treatment with P1446A failed to induce an extensive unfolded protein response. This study provides rationale for additional investigations of P1446A in CLL.

## Introduction

Over 15,000 people are diagnosed with chronic lymphocytic leukemia (CLL) in the United States each year with a 50–70% decrease in 10-year survival compared to the general population [1]. Novel therapies which target B-cell receptor (BCR) signaling, such as an oral Bruton’s tyrosine kinase (BTK) inhibitor ibrutinib, demonstrate impressive activity in treatment of CLL [2]. However, acquired mutations in BTK account for ibrutinib resistance [3]. Furthermore, novel agents are less efficacious in high-risk CLL, including those with TP53 abnormalities, which remains an unmet clinical need [4, 5].

Cyclin-dependent kinases (CDKs) are a class of serine/threonine kinases that regulate the cell cycle and are responsible for its orderly progression. Association of a CDK catalytic subunit with a regulatory Cyclin subunit results in an activated Cyclin-CDK protein complex. The activity of distinct Cyclin-CDK complexes fluctuates throughout the cell cycle, where they play unique roles due to relative substrate specificity [6, 7]. Since the majority of CLL cells rest in G_0_, accounting for low clonal turnover [8], they are resistant to selective inhibition of mitotic (CDK1) and interphase (CDK2/4/6) kinases. By contrast, CLL is thought to be primarily a disease of defective B-cell apoptosis. CLL cells express most pro-survival proteins of the BCL2 family (e.g., BCL2, MCL1, BFL1). The balance between them and the pro-apoptotic BCL2 family proteins (BIM, NOXA, PUMA) determines cell fate and accounts for apoptosis resistance [9]. Due to their short half-life, expression of some anti-apoptotic proteins is dependent on unperturbed function of RNA polymerase II (RNAPII). The latter is regulated by the transcriptional kinases, of which CDK7/9 are the best studied. CDK7 and cyclin H are components of the general transcription factor TFIIH involved in transcription initiation [10]. CDK9/cyclin T complex forms the catalytic core of the positive transcription elongation factor b (pTEFb) and is critical for stimulation of transcription elongation [11, 12]. Inhibition of CDK7/9 results in reduced global transcription and decreased synthesis of short-lived anti-apoptotic proteins such as MCL1, thus promoting apoptosis [13–16]. Furthermore, it is now being recognized that effects not related to transcriptional regulation contribute to the CDK inhibitor-mediated cytotoxicity in CLL, such as induction of endoplasmic reticulum (ER) stress and the unfolded protein response (UPR) [17].

While a number of CDK inhibitors showed promise in the pre-clinical setting [13, 18, 19], flavopiridol and dinaciclib have been most extensively studied in clinical trials in CLL. Remarkably, both agents were active in high-risk CLL. The pan-CDK inhibitor flavopiridol (HMR-1275, alvocidib, Sanofi) led to cell cycle arrest and inhibited CDK9 in HeLa cells *in vitro* [20] and induced responses in 53% of patients with relapsed/refractory CLL [21]. Dinaciclib (SCH-727965, Merck) an inhibitor of CDK1/2/5/9 [22], showed an overall response rate of 54% in the same group of patients [23]. However, due to a high frequency of adverse events, including severe tumor lysis syndrome, novel CDK inhibitors are needed to improve therapeutic strategies in CLL.

P1446A-05 (hereby referred to as P1446A, Piramal Healthcare Ltd.) is a novel investigational CDK inhibitor. P1446A inhibits CDK1, 2, 4, 5, 6, 8 and 9 with IC_50_ values of at 25, 180, 90, 210, 210, 12 and 22 nmol/L, respectively (S1 Fig). P1446A induced cell cycle arrest and apoptosis of solid tumor cell lines *in vitro* and restricted growth in human colon (HCT-116) and non-small cell lung (H-460) carcinoma xenograft mouse models (Piramal Healthcare Ltd., proprietary information; [24]). Furthermore, P1446A showed promising activity in two independent Phase I clinical trials in patients with solid tumors [25, 26]. Here we demonstrate that P1446A inhibits CDK activity and induces rapid apoptosis in peripheral blood CLL cells. The cytotoxic effect of P1446A is dependent upon activation of apoptosis stress-inducing kinase 1 (ASK1), mitogen activated protein kinases (MAPK) c-Jun N-terminal kinase (JNK; MAPK8) and p38 MAPK, and subsequent induction of NOXA, a pro-apoptotic BCL2 family protein.

## Methods

### Patient samples, CLL and stromal cell co-cultures

Following approval by the Committee for Protection of Human Subjects at Dartmouth-Hitchcock Medical Center, the Institutional Review Board at Oregon Health and Science University and written consent of patients, peripheral blood was obtained from patients with B-CLL at the hematology clinics at the above institutions (N = 52). The mean time from CLL diagnosis to entry of study was 5 years; 39 patients (75%) were previously untreated. Isolation of peripheral blood mononuclear cells (PMBCs) was performed using standard Ficoll-Hypaque technique (Amersham, Piscataway, NJ). CLL samples had more than 90% CD5^+^/CD19^+^ cells, as determined by flow cytometry. CLL cells were cultured in RMPI-1640 medium supplemented with 15% fetal bovine serum, 100 U/mL penicillin, 100 μg/mL streptomycin, 2 mM L-glutamine, 25 mM HEPES, 100 μM non-essential amino acids and 1 mM sodium pyruvate (Lonza, Walkersville, MD). 10 CLL samples with 17p deletion were obtained from the CLL Center at Dana-Farber Cancer Institute. All experiments were performed with freshly isolated cells except the viability assays involving the latter, which were performed with viably frozen cells.

Mouse fibroblast cell line (L cells) engineered to express CD40L (L4.5) was kindly provided by Dr. Sonia Neron (Hema-Quebec, Quebec, Canada) [27]. Parental L cells were obtained from American Type Culture Collection (Manassas, VA). All were maintained in RPMI 1640 medium with 10% FBS and penicillin-streptomycin. CLL cells were cultured under standardized condition on stroma as previously described [28]. Cultures were then treated with drugs for the indicated time periods.

### Cell viability testing and drugs

CLL cell apoptosis was measured in duplicates as previously described using the ApoScreen Annexin V Apoptosis Kit [29]. Briefly, cells were resuspended in 150 μl of Annexin V binding buffer containing 1 μl of Annexin V-PE, 1 μl of 7-aminoactinomycin D (7-AAD) and 1 μl of CD19-mAbs (Southern Biotech, Birmingham, AL) followed by flow cytometry on a MACSQuant flow cytometer (Miltenyi Biotec, San Diego, CA). Flow cytometry analysis was performed using FlowJo software (Tree Star, Ashland, OR). P1446A was provided by Piramal Healthcare Ltd. (Mumbai, India). JNK Inhibitor VIII, SP600125, SB203580, ABT-737 and PD0332991 were obtained from Selleck Chemicals (Houston, TX) and CVT-313—from Santa Cruz Biotechnology (Dallas, TX).

### Immunoblotting

Cells were lysed in radioimmunoprecipitation (RIPA) buffer (20 mM Tris, 150 mM NaCl, 1% NP-40, 1 mM NaF, 1 mM Sodium phosphate, 1 mM NaVO3, 1 mM EDTA, 1 mM EGTA), supplemented with protease inhibitor cocktail (Roche, Indianapolis, IN), phosphatase inhibitor cocktail 2 (Sigma-Aldrich) and 1 mM phenylmethanesulfonyl fluoride (PMSF). Proteins were analyzed by immunoblotting as previously described [29]. The following antibodies were used: phospho-ASK1 (Ser83), phospho-ASK1 (Thr845), ASK1, phospho-ATF2 (Thr67/71), phospho-Bad (Ser112), BCL2, BIM, phosopho-eIF2α (Ser51), GAPDH, JNK, phospho-JNK (Thr183/Tyr185), p38 MAPK, phospho-p38 MAPK (Thr180/Tyr182), MCL1, PARP and cleaved PARP, PERK, phospho-PRAS40 (Thr246), phospho-Rb (Ser780), XIAP (Cell Signaling Technology, Danvers, MA), β-actin (Sigma-Aldrich), Rb and ATF4 (Santa Cruz Biotechnology), phospho-GSK3β (Tyr216) (Abcam, Cambridge, MA), Noxa (Novus Biologicals, San Diego, CA), phospho-Rb (Thr821) (Life Technologies, Camarillo, CA), RNAPII, phospho-RNAPII (Ser2), phospho-RNAPII (Ser5) (Covance, Princeton, NJ), and horseradish peroxidase-conjugated anti-mouse and anti-rabbit antibodies (BioRad). Densitometry was performed using ImageJ software (Bethesda, MD).

### Quantitative Real-Time PCR

Total RNA was isolated using the RNeasy Mini Kit (Qiagen, Hilden, Germany). cDNA was synthesized from 500 ng of RNA using the iScript cDNA Synthesis Kit (Bio-Rad, Hercules, CA). Quantitative real-time PCR (RT-PCR) was performed in a C1000 Thermal Cycler (Bio-Rad) using Universal PCR Master Mix according to the manufacturer’s instructions (Applied Biosystems, Grand Island, NY) with template cDNA and gene-specific probes. The following probes were used: NOXA, Hs00560402_m1; PUMA, Hs00248075_s1; MCL1, Hs01050896_m1. Amplification of the sequence of interest was compared to a reference probe (GAPDH, Hs02758991_g1, all from Life Technologies). All samples were analyzed in duplicate. We used the comparative C_t_ method for relative quantitation (2^-ΔΔCt^, where ΔΔC_t_ = ΔC_tP_− ΔC_tK_; P = probe and K = reference sample). XBP1 spiced and unspliced variants were detected using the following primers: 5’-GTTGAGAACCAGGAGTTAAGACAG-3’ (forward) and 5’-CAGAGGGTATCTCAAGACTAGG-3’ (reverse).

### siRNA-mediated gene silencing

Electroporation of siRNA into CLL cells was performed using Amaxa Human B-cell Nucleofection Kit (Amaxa, Cologne, Germany). 1x10^7^ PBMCs were mixed with 100 μL of Amaxa B-cell nucleofector solution, and 2 μg of siRNA was nucleofected using program X-005 [29]. Transfection efficiency, assessed by transfection with 2 μg pMaxGFP plasmid, was 30–60% with cell viability of 50–80% at 24 hours. siRNA oligos were synthesized by Dharmacon (Lafayette, CO). Sense strand against human ASK1: GCUCGUAAUUUAUACACUGUU.

### IGHV Mutation status

Following DNA isolation using the Qiagen DNeasy Blood and Tissue Kit, *IGHV* mutations were analyzed using the IGH Somatic Hypermutation Assay v2.0 (Invivoscribe, San Diego, CA). PCR was performed using the supplied master mixes and Amplitaq Gold DNA polymerase (Applied Biosystems) on a Veriti Thermal Cycler Model #9902 (Applied Biosystems) to amplify the IGH sequence fragment between the leader (VHL) and joining (J) regions as per the manufacturer's instructions. To confirm amplification of a single clonal product in the expected size range, an aliquot of each sample was run on a 1.5% agarose gel and separated by gel electrophoresis. Samples were then submitted for DNA sequencing using the supplied sequencing primers. The NCBI IgBLAST tool was used to determine the percent divergence of each clonal sequence. Samples that showed less than 2% divergence from germline sequence were deemed to have un-mutated *IGHV*.

### Statistical analysis

Statistical analysis was performed with Student t test and Wilcoxon signed-rank test in GraphPad Prism software (LaJolla, CA). p<0.05 was considered to be statistically significant. Fisher exact test was used to identify ontology groups and pathways statistically enriched in the gene set.

## Results

### P1446A induces apoptosis in primary CLL cells

**Fig 1:**
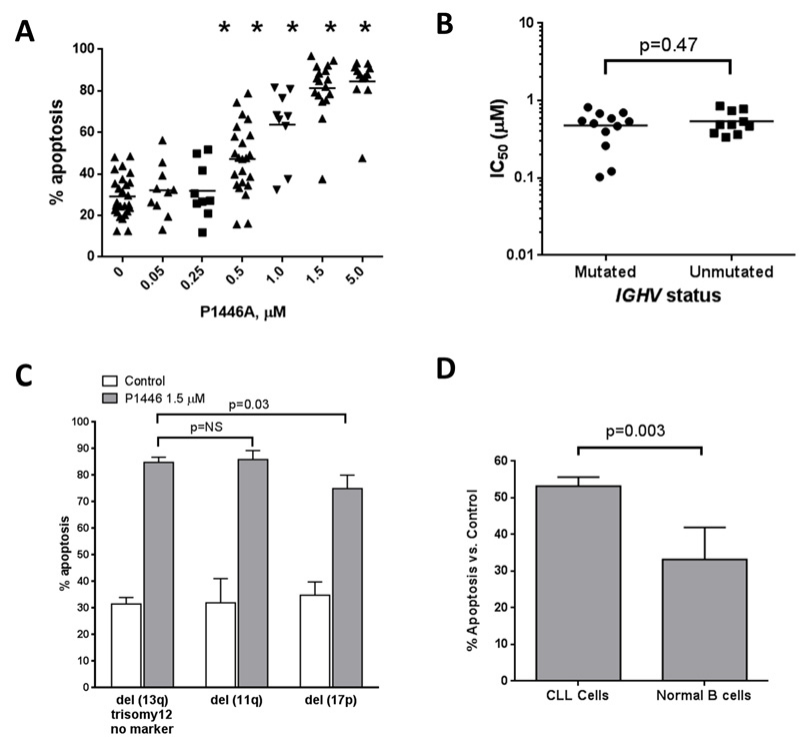
CDK inhibitor P1446A induces apoptosis of the CLL B-cells independent of *IGHV* mutational status and cytogenetics. *(A)* PBMC's from patients with CLL (N = 10) were incubated with 0.05–5 μM P1446A or vehicle control for 24 hours and assayed for apoptosis. Here and subsequently, apoptosis was determined by Annexin V staining within the CD19^+^ subset of cells. Horizontal lines represent the mean. *(B)* CLL B-cells were incubated with 0.05–1.5 μM P1446A for 24 hours. IC_50_ value was determined for CLL samples with mutated (N = 13) and unmutated *IGHV* (N = 10). *(C)* CLL B-cells (N = 62) were incubated with 1.5 μM P1446A for 24 hours. Cytogenetics markers were determined by fluorescent in situ hybridization [no marker, 11q, trisomy 12, 13q, 17p]. Horizontal lines represent the mean. *(D)* PBMCs from patients with CLL (N = 37) or healthy volunteers (N = 6) were incubated with 1.5 μM P1446A or vehicle control for 24 hours. Since we noted a significant variation in baseline apoptosis between patient samples, normalization to the time-matched untreated controls was performed to more clearly reflect the drug-induced apoptosis. *- p<0.05 compared to untreated control.

Since CDK inhibitors are directly cytotoxic to neoplastic B-cells in vitro [13, 19, 30], we first investigated whether P1446A induced apoptosis in primary CLL cells obtained from peripheral blood. Upon 24 hour incubation, cell death was evident at a concentration of 0.5 μM P1446A: 47.2±3.5% cells underwent apoptosis compared to 29.2±2.0% cell treated with vehicle control (Fig 1A), as previously reported by us at the American Society of Hematology Annual Meeting [31]. Treatment with 1.5 μM P1446A resulted in almost complete apoptosis of CLL cells (83.3±3.3%) and further increases in drug concentrations did not lead to additional toxicity. CLL cell apoptosis did not correlate with individual disease characteristics, such as *IGHV* mutational status, expression of CD38 and ZAP-70 (Fig 1B and data not shown). CLL samples manifesting high-risk cytogenetic abnormalities similarly underwent apoptosis upon treatment with P1446A, albeit CLL samples with *TP53* aberrations showed a slightly decreased susceptibility (Fig 1C). Compared with normal donor B-cells, CLL cells were more susceptible to P1446A-induced apoptosis (Fig 1D).

**Fig 2:**
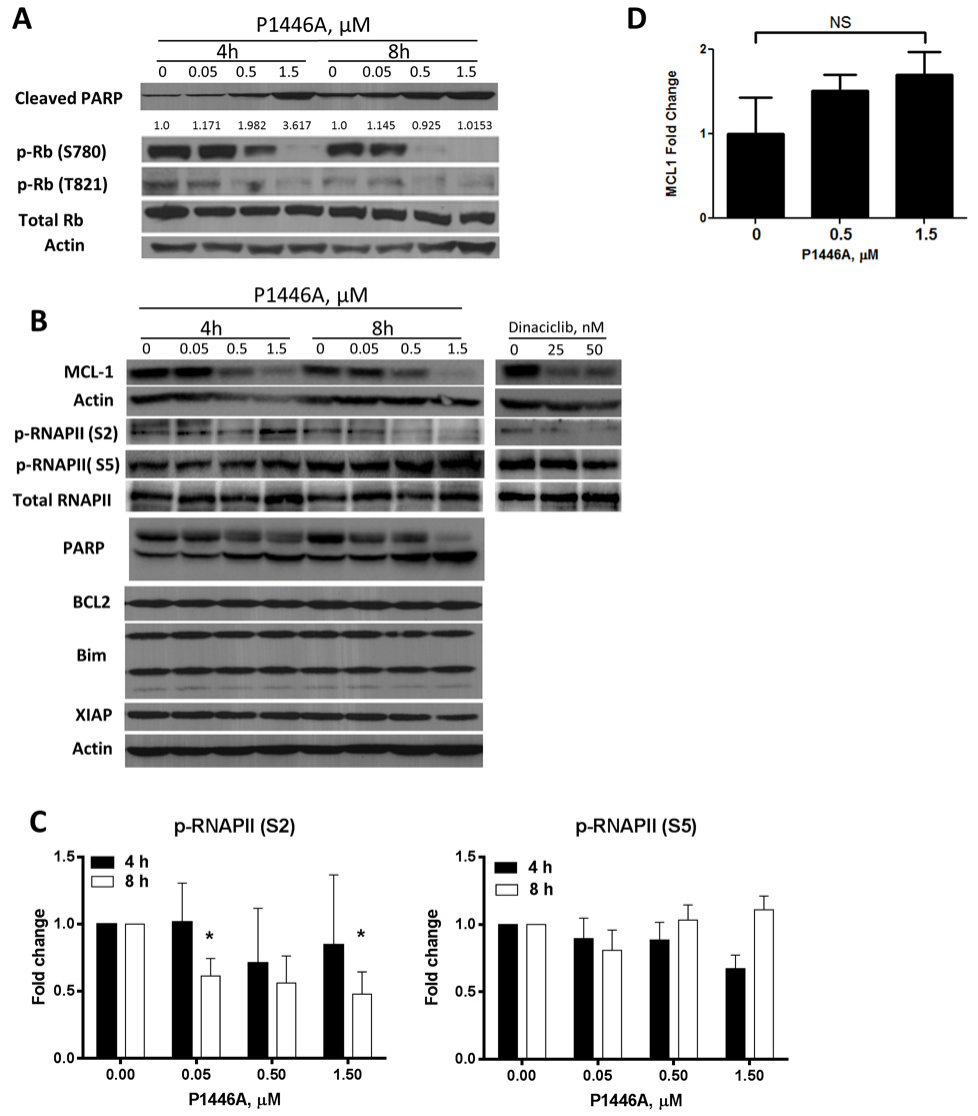
P1446A inhibits interphase CDKs in CLL B-cells. *(A-C)* CLL cells were incubated with P1446A at the indicated concentrations for 4 or 8 hours. As a control, cells were incubated with dinaciclib for 4 hours. Whole-cell protein lysates were subjected to immunoblotting. Representative images and densitometry data from four individual patients are shown. *- p<0.05 compared to untreated control. (*D*) CLL cells from 6 individual patients were incubated with 0–1.5 μM P1446A for 6 hours. Total RNA was isolated from CD19^+^ CLL B-cells, reverse-transcribed and subjected to real-time PCR with the indicated probes (in duplicates). Results were normalized to 18S levels. Data are the mean ± SE.

To measure the effect of P1446A on interphase CDKs (CDK2, 4, and 6), we studied changes in the retinoblastoma (Rb) protein phosphorylation. Since CLL cell viability was impaired at early timepoints as evidenced by PARP cleavage, we incubated cells for 4 and 8 hours (Fig 2A and 2B). Treatment of CLL cells with P1446A resulted in reduced Rb phosphorylation (Rb^T821^ and Rb^S780^), suggesting that interphase CDKs were inhibited [32–34]. Since peripheral blood CLL cells rest in G_0_, one would predict that disruption of CDK activity would not affect cell viability. Consistent with that notion, use of selective inhibitors of CDK2 (CVT-313) or CDK4/6 (PD0332991) alone or in combination did not lead to CLL cell apoptosis (data not shown). Interestingly, we did not see an effect of P1446A on RNAPII phosphorylation at either Ser5 (CDK7-specific site) or Ser2 (CDK9-specific site) after 4 hours incubation with the drug, indicating that RNAPII function was not fully inhibited prior to apoptosis initiation (Fig 2B and 2C). A partial reduction in phosphorylation at Ser2 site was observed after an 8-hour incubation with P1446A. At the same time, treatment with P1446A resulted in downregulation of MCL1, but not BCL2 or X-linked inhibitor of apoptosis (XIAP; Fig 2B). MCL1 downregulation could be due to rapid protein turnover. Since treatment with P1446A did not significantly decrease MCL1 mRNA levels in CLL cells (Fig 2D), enhanced MCL1 degradation may have accounted for its downregulation. We also treated CLL cells with dinaciclib as a control and observed rapid loss of phosphorylation of RNAP at S2 accompanied by decreased expression of MCL1 (Fig 2B). Meanwhile, anti-apoptotic BCL2 family proteins BCLX and BFL1 were not present at detectable levels in peripheral blood CLL cells [28] and thus were not analyzed in this experiment. These data implicate that disruption of interphase or transcriptional CDKs may not be sufficient for apoptosis induction by P1446A, and additional mechanisms may be responsible for the effects of this agent in CLL.

Because of these findings, we investigated whether P1446A could target other kinases in CLL. Glycogen synthase 3ß (GSK3ß) as well as PIM kinase isoforms were shown to be targeted by P1446A *in vitro* in the nanomolar concentration range (S1 Fig) and were both implicated in cancer and CLL [35–38]. GSK3ß phosphorylates ß-catenin at Ser33, 37, and 41 to target it for ubiquitylation and subsequent degradation [38]. We did not observe either a reduction in GSK3ß phosphorylation at Tyr216 (its auto-phosphorylation site) or accumulation of ß-catenin upon CLL cell incubation with P1446A (S2A Fig). Similarly, PIM kinases were not inhibited as evidenced by lack of an effect on its downstream targets (S2B Fig).

In summary, P1446A rapidly induced apoptosis in CLL cells, and this effect was independent of inhibition of transcriptional CDKs.

### P1446A activates JNK/p38 MAPK in CLL

The above observation that P1446A induces apoptosis in primary CLL cells while transcriptional CDKs are not inhibited led us to explore alternative mechanisms of drug-mediated cell death. Flavopiridol, a pan-CDK inhibitor, was previously reported to induce ER stress and the unfolded protein response (UPR) [17], a cell signaling process poised to correct misfolded proteins within the ER lumen following ER stress [39, 40]. The UPR is heralded by the activation of at least three major stress sensors: protein kinase RNA-like ER kinase (PERK), inositol-requiring enzyme 1 (IRE1), and activating transcription factor 6 (ATF6) [41]. PERK activation leads to phosphorylation of the α-subunit of eukaryotic translation initiator factor 2α (eIF2α), halting translation but allowing synthesis of ATF4 [39]. Meanwhile, dimerization and autophosphorylation of IRE1 triggers production of a spliced variant of X-box binding protein 1 (XBP1), which acts as a transcription factor. Finally, ATF6 is transported to the Golgi where it is modified to control genes encoding ER-associated degradation (ERAD) components as well as regulate XBP1 activity [39, 40]. Together, these processes govern protein folding in the ER.

**Fig 3:**
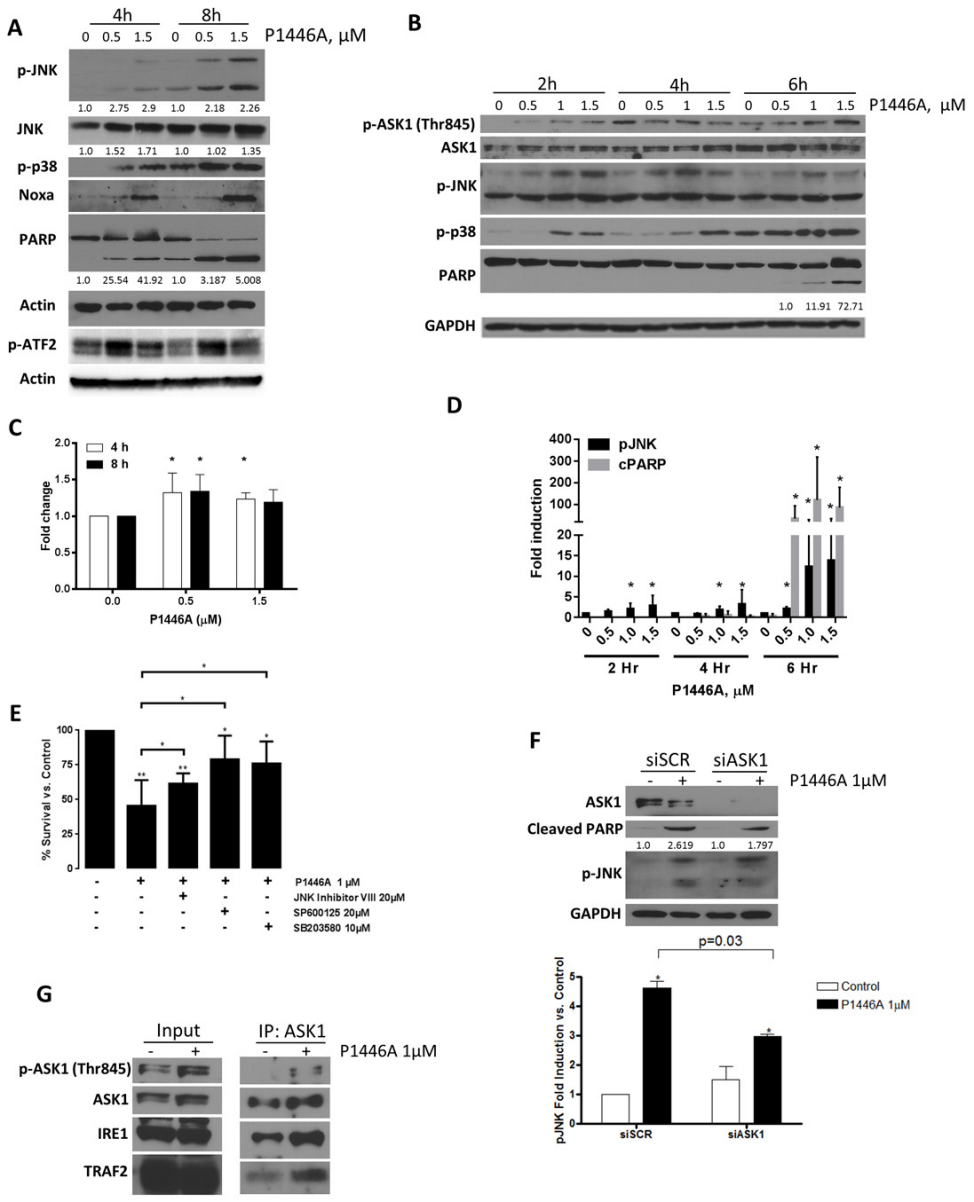
P1446A induces JNK and p38 MAPK in CLL cells. *(A-D)* CLL cells obtained from four individual patients were incubated with P1446A (0.5–1.5 μM) for 4 or 8 hours. Whole-cell protein lysates were subjected to immunoblotting. Representative images and densitometry data are shown. For densitometry, a fold increase in expression of pATF2 (C) and pJNK/cPARP (D) referenced to total levels of protein at each dose and time point were normalized to their expression in untreated samples *(E)* CLL cells obtained from six individual patients were incubated with 20 μM JNK inhibitor VIII, 20 μM SP600125, 10 μM SB203580 or vehicle control for 1 hour and then with 1 μM P1446A for 24 hours. Apoptosis was determined by Annexin V and 7-AAD staining within the CD19^+^ subset of cells. *–p<0.05; **—p<0.01 vs. control. *(F)* CLL cells were transfected with siRNA's against ASK1 or control siRNA using Amaxa program X-05 and subsequently incubated with 1 μM P1446A or vehicle control for 24 h. Whole-cell lysates were subjected to immunoblotting. A representative blot of four independent experiments is shown, along with densitometry data (*- p<0.05 vs. control). *(G)* CLL cells were incubated with 1 μM P1446A or vehicle control for 6 hours. Protein lysates were incubated with antibody against ASK1 or isotype control (not shown) and subjected to immunoblotting. A representative blot of three independent experiments is shown.

Importantly, flavopiridol was shown to induce IRE1 to complex with and activate ASK1, and subsequently JNK/p38 MAPK in CLL B-cells *in vitro* and *in vivo* [17]. Given these data, we supposed that P1446A could lead to ER stress induction in primary CLL cells, culminating in apoptosis. We noted rapid activation of JNK and p38 MAPK in CLL cells treated with P1446A *in vitro*. Phosphorylation of JNK and p38 MAPK occurred within 2 hours of treatment with P1446A and preceded apoptosis as measured by PARP cleavage (Fig 3A–3D). To determine if JNK/p38 MAPK activation was a key event in P1446A-induced apoptosis, CLL cells were treated with corresponding pharmacologic inhibitors, resulting in partial protection from cell death (Fig 3E). Transcription factors c-Jun and activating transcription factor 2 (ATF2) are well-recognized downstream targets of JNK and p38 MAPK. While, unexpectedly, c-Jun was not phosphorylated in response to treatment with P1446A (data not shown), we observed activation of ATF2 (Fig 3A and 3C).

Subsequently, we sought to identify the upstream regulators of JNK in this setting and found rapid phosphorylation of ASK1 at Thr845, a site required for kinase activity, in CLL cells treated with P1446A. ASK1 activation occurred in a time and dose-dependent manner and was concurrent with JNK phosphorylation (Fig 3B). Meanwhile, siRNA-mediated knockdown of ASK1 led to a reduction in JNK phosphorylation and a partial rescue from apoptosis (Fig 3F). ASK1 forms a trimeric complex with IRE1 and tumor necrosis factor receptor-associated factor 2 (TRAF2) to induce the MAPK signaling cascade and apoptosis in CLL [39, 42–44]. Using immunoprecipitation technique, we observed formation of the trimeric complex between ASK1, IRE1, and TRAF2 (Fig 3G). This strongly suggests that treatment with P1446A leads to rapid ASK1 activation in CLL with subsequent phosphorylation of stress kinases JNK and p38 MAPK, ultimately leading to cell apoptosis.

### P1446A does not induce the common UPR pathways in CLL cells

**Fig 4:**
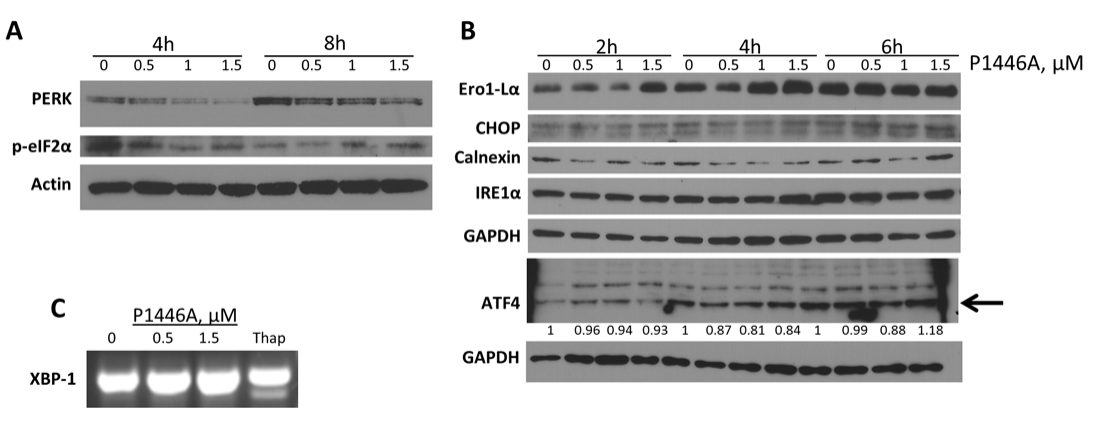
The UPR in response to P1446A treatment is limited. *(A*, *B)* CLL cells obtained from four individual patients were incubated with P1446A (0.5–1.5 μM) for the indicated time intervals. Whole-cell protein lysates were subjected to immunoblotting. Representative images from 1 of 3 independent experiments are shown. *(C)* Cells were incubated with the indicated concentration of P1446A or 1 μM thapsigargin (thap) for 6 hours, total RNA was isolated, reverse-transcribed and subjected to PCR using XBP1-specific primers.

Subsequently, we analyzed whether additional pathways within the UPR were activated in response to treatment with P1446A. However, we did not detect activation of the three main stress sensors or their downstream targets in CLL cells (Fig 4). Specifically, hyper-phosphorylation of PERK (as would be manifested by mobility shift) and ATF4 induction were not observed (Fig 4A and 4B). Meanwhile, phosphorylation of PERK substrate eIF2α was not affected in a consistent fashion upon treatment of CLL cells with P1446A (Fig 4A). Similar to the effects of flavopiridol, we did not detect either changes in IRE1α or XBP1 splicing in CLL cells treated with P1446A (Fig 4B and 4C). To further explore potential consequences of ER stress, we quantified other targets involved in the UPR and ERAD, and did not observe a change in Ero1-Lα, CHOP and calnexin proteins (Fig 4B). Thus, we did not detect evidence of the UPR beyond ASK1 activation in CLL cells treated with P1446A *in vitro*.

### Upregulation of NOXA is a result of P1446A-mediated JNK activation in CLL

**Fig 5:**
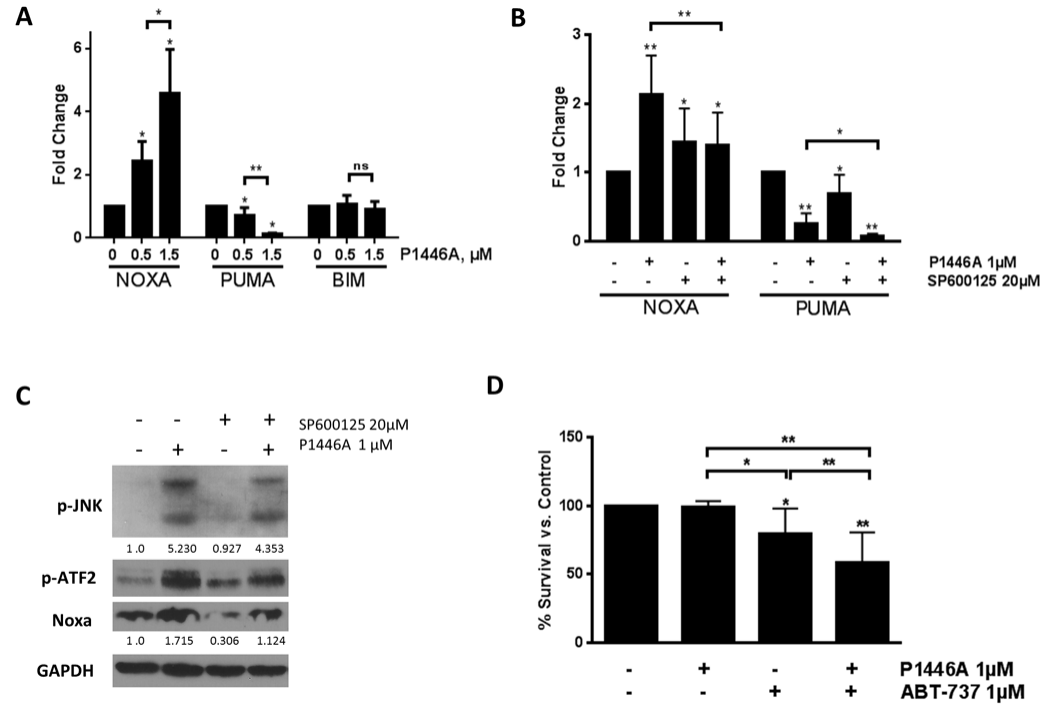
P1446A induces NOXA and cooperates with ABT-737 to reverse apoptosis resistance. *(A-B)* CLL cells from 6 individual patients were incubated with the indicated doses of P1446A for 6 hours with or without 20 μM SP600125. Total RNA was isolated from CD19^+^ CLL B-cells, reverse-transcribed and subjected to real-time PCR with the indicated probes (in duplicates). Results were normalized to 18S levels. Data are the mean ± SE. *(C)* CLL cells (N = 3) were incubated with 20 μM SP600125 or vehicle control for 1 hour and then with 1 μM P1446A for 6 hours. Whole-cell protein lysates were subjected to immunoblotting. *(D)* CLL cells (N = 6) were co-cultured with CD40L-expressing stroma for 24 hours and subsequently incubated with the indicated doses of P1446A and ABT-737 or vehicle control for 24 hours. Apoptosis was determined by Annexin V and 7-AAD staining within the CD19^+^ subset of cells. **—p<0.01 vs. control.

JNK regulates NOXA, a pro-apoptotic BH3-only protein, an effect likely mediated through the ATFs [45]. In accordance with that, we found that treatment with P1446A led to a dramatic induction of NOXA mRNA transcript and protein levels (Figs 3A and 5A). By contrast, mRNA transcript levels of the BH3-only protein PUMA were downregulated following P1446A treatment, while BIM mRNA and protein expression remained stable (Figs 5A and 2B). Pre-treatment of the CLL cells with JNK inhibitor SP600125 abrogated the P1446A-mediated effects on NOXA, while PUMA transcript levels were not affected (Fig 5B and 5C), suggesting that NOXA upregulation is JNK-dependent in this setting.

We and others have previously reported that CD40 ligation leads to robust upregulation of the anti-apoptotic BCL2 family proteins BCLX and BFL1 and induces drug resistance in CLL cells [28, 46, 47]. Because NOXA specifically interacts with MCL1 but not BCLX, CLL cell co-culture with CD40L-expressing stromal cells induced resistance to P1446A (Fig 5D). However, combination of P1446A with the BH3-mimetic ABT-737, a BCL2/X inhibitor, partially reversed CD40L-mediated protection of CLL cells (Fig 5D).

## Discussion

CDK inhibitors show promise in the therapy of B-cell malignancies where their efficacy has been attributed to inhibition of transcriptional CDKs with subsequent downregulation of short-lived pro-survival proteins, such as MCL1 [13, 15, 48]. However, it is now being recognized that CDK inhibitors exert their anti-tumor effect in CLL through several additional mechanisms. For example, CDK inhibitors induce ER stress and the UPR, inhibit autophagy and abrogate the canonical NFκB pathway via regulation of the IκB kinase complex [17, 18].

The UPR is a series of adaptive mechanisms activated upon ER stress which cope with protein-folding alterations in the cell. However if ER stress becomes severe and irreversible, the UPR eliminates damaged cells by apoptosis [41]. Pharmacologic agents can induce ER stress and the UPR in CLL cells, however the extent of the UPR may be limited in neoplastic B-cells. A CDK inhibitor flavopiridol was shown to induce partial ER stress response in CLL cells both *in vitro* and *in vivo*, which included nuclear translocation of ATF6 and an increase in XBP1, IRE1 and GRP78 mRNA expression. Meanwhile, activation of either PERK or IRE1, XBP1 splicing and CHOP induction were not observed [17]. By contrast, CLL cells treated with auranofin demonstrated induction of both GRP78 and CHOP proteins [49]. In cell lines, including a prolymphocytic leukemia cell line MEC1, both agents as well as thapsigargin induced robust ER stress response which included PERK phosphorylation, XBP1 splicing and CHOP induction, indicating that ER stress response is dysfunctional in CLL [17, 49]. The UPR, manifested by an increased expression of CHOP, GRP78 and XBP1, can also be activated in response to BCR stimulation in CLL cells [50]. Yet, in addition to substantial variability between individual samples, CLL cells showed only weak activation of the PERK arm, and no IRE1-dependent processing of XBP1 following IgM cross-linking [50].

There are several components of the UPR that could contribute to ER-stress mediated apoptosis. The ASK1-dependent apoptotic pathway is triggered following complex formation between IRE1, the adaptor molecule TRAF2 and the MAPK pathway member ASK1 [51]. ASK1 was activated in CLL cells treated with flavopiridol *in vitro* as well as *in vivo* (from patients who received flavopiridol, thus indicating relevance of these findings to the clinic) [17]. Here we demonstrate that P1446A acts in a manner similar to flavopiridol in CLL cells, inducing trimerization of ASK1 with IRE1 and TRAF2. This results in phosphorylation of stress-activated protein kinases JNK and p38 MAPK–key signaling events accounting for P1446A-mediated toxicity in CLL—which have also been previously linked to ER stress and the UPR [52].

While P1446A inhibits interphase CDKs, quiescent CLL cells are not dependent on their activity and are resistant to selective inhibition of CDK2 and CDK4/6. Additionally, functional overlap between cell cycle-regulating CDKs may further render cells resistant to selective inhibition of individual kinases [53]. Furthermore, P1446A-induced apoptosis did not fully rely on inhibition of transcriptional CDKs (7/9). Similar to findings with flavopiridol, neither PERK activation nor XBP1 processing occurred in CLL cells treated with P1446A, further strengthening the hypothesis that ER stress is dysfunctional in primary neoplastic B-cells. However, we observed robust induction of BH3-only protein NOXA. This followed JNK activation in CLL cells treated with P1446A, likely mediated via ATF2/3. Upregulation of the anti-apoptotic proteins not targeted by NOXA, such as BCLX, in response to CD40 signaling, rescued CLL cells from P1446A-mediated death, confirming the critical role of NOXA to P1446A-mediated apoptosis. As previously reported by our group, other drugs, such as the microtubule-disrupting agents (vincristine) induce JNK and NOXA in CLL cells *in vitro* and *in vivo* resulting in apoptosis [54, 55]. A distinct mechanism which involves JNK-dependent proteasomal degradation of MCL1 could be responsible for apoptosis induction in this setting [56]. Since both JNK and p38 MAPK phosphorylate MCL1, and target it for proteasomal degradation [56, 57], it is conceivable that along with NOXA induction, this may represent an additional mechanism of P1446A toxicity in CLL. Further investigations of the role of the individual components of the UPR in P1446A-mediated CLL cell apoptosis are necessary, but will be limited by technical challenges of disrupting those components in CLL cells.

In addition to interphase and transcriptional CDKs, P1446A is also known to inhibit the atypical cyclin-dependent kinase CDK5 *in vitro* (at 210 nM). Unlike other CDKs who partner with cyclins, CDK5 binds the regulatory subunits p35 and p39. Upon activation, p35 is proteolytically processed to the p25 subunit, which then augments CDK5 activity [58]. CDK5 has been classically associated with neuronal development and neurogenerative disease [59]. However, recent reports demonstrate that CDK5 takes part in the stress response to reactive oxygen species and participates in NOXA regulation in both neural and cancer cell types [60, 61]. In CLL, genetic knockdown of CDK5 has been associated with ER stress response similar to flavopiridol treatments [17], and hence its inhibition may play a role in P1446A-mediated JNK/p38 MAPK activation and death. Additional experiments would be necessary to verify the role of CDK5 in response to P1446A and other CDK inhibitors in CLL.

In summary, we demonstrate that CDK inhibitor P1446A is a potent inducer of apoptosis in primary CLL cells in vitro. P1446A leads to partial activation of ER stress and the UPR in CLL cells manifested by ASK1-dependent signaling, leading to JNK/p38 MAPK activation and up-regulation of Noxa. This study provides rationale for additional investigations of P1446A in CLL and B-cell malignancies, both alone and in combination with other novel agents, like venetoclax (ABT-199).

## Supporting Information

S1 Fig Determination of selectivity of P1446A-05 was established using biochemical kinase profiling (Kinase 400-Profiler, ProQinase, Freiburg, Germany). IC_50_ values were determined for the 37 kinases whose activity was inhibited by ≥60% at a drug concentration of 10 μM or less (as shown). 19 kinases were potently inhibited by P1446A (IC50 <0.5 μM).
(PPTX)

S2 Fig P1446A does not inhibit activity of GSK3ß or PIM kinases in CLL. CLL cells were incubated with P1446A over the indicated time period. Whole-cell protein lysates were subjected to immunoblotting. Representative blots from 1 of 3 independent experiments are shown.
(PPTX)
