# Supplementary figures and images for: Cyclin-Dependent Kinase Inhibitor P1446A Induces Apoptosis in a JNK/p38 MAPK-Dependent Manner in Chronic Lymphocytic Leukemia B-Cells
Source: PLoS One. 2015 Nov 25;10(11):e0143685. doi: 10.1371/journal.pone.0143685 (PMC4659573; doi:10.1371/journal.pone.0143685)

## Slide 1
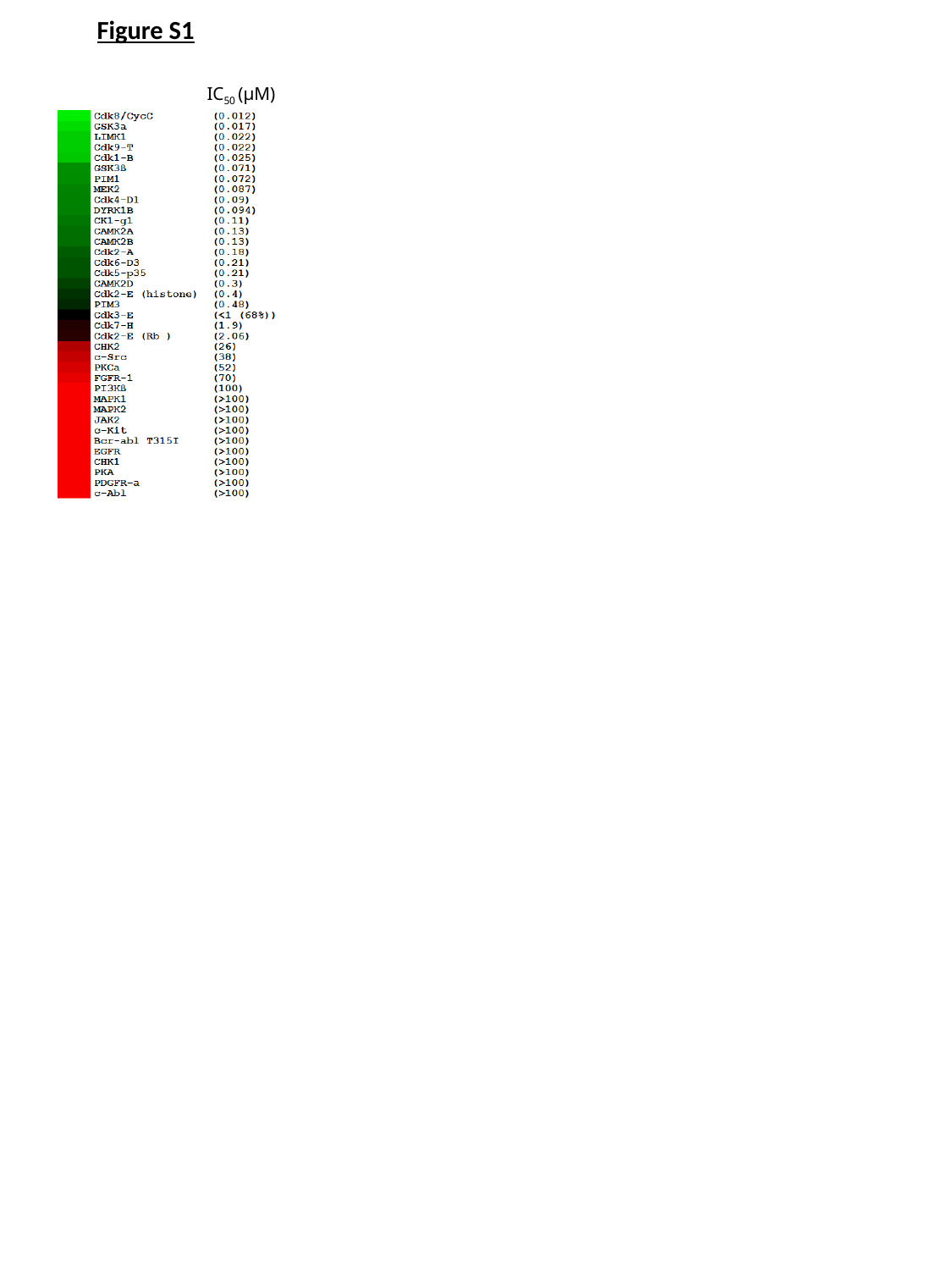

# Figure S1
IC50 (µM)

Supplement: S1 Fig — IC50 values were determined for the 37 kinases whose activity was inhibited by ≥60% at a drug concentration of 10 μM or less (as shown). 19 kinases were potently inhibited by P1446A (IC50 <0.5 μM). (PPTX) [file pone.0143685.s001.pptx]
