# Supplementary material for: Cyclin-Dependent Kinase Inhibitor P1446A Induces Apoptosis in a JNK/p38 MAPK-Dependent Manner in Chronic Lymphocytic Leukemia B-Cells
Source: PLoS One. 2015 Nov 25;10(11):e0143685. doi: 10.1371/journal.pone.0143685 (PMC4659573; doi:10.1371/journal.pone.0143685)
Supplement: S2 Fig — CLL cells were incubated with P1446A over the indicated time period. Whole-cell protein lysates were subjected to immunoblotting. Representative blots from 1 of 3 independent experiments are shown. (PPTX) [file pone.0143685.s002.pptx]

## Slide 1
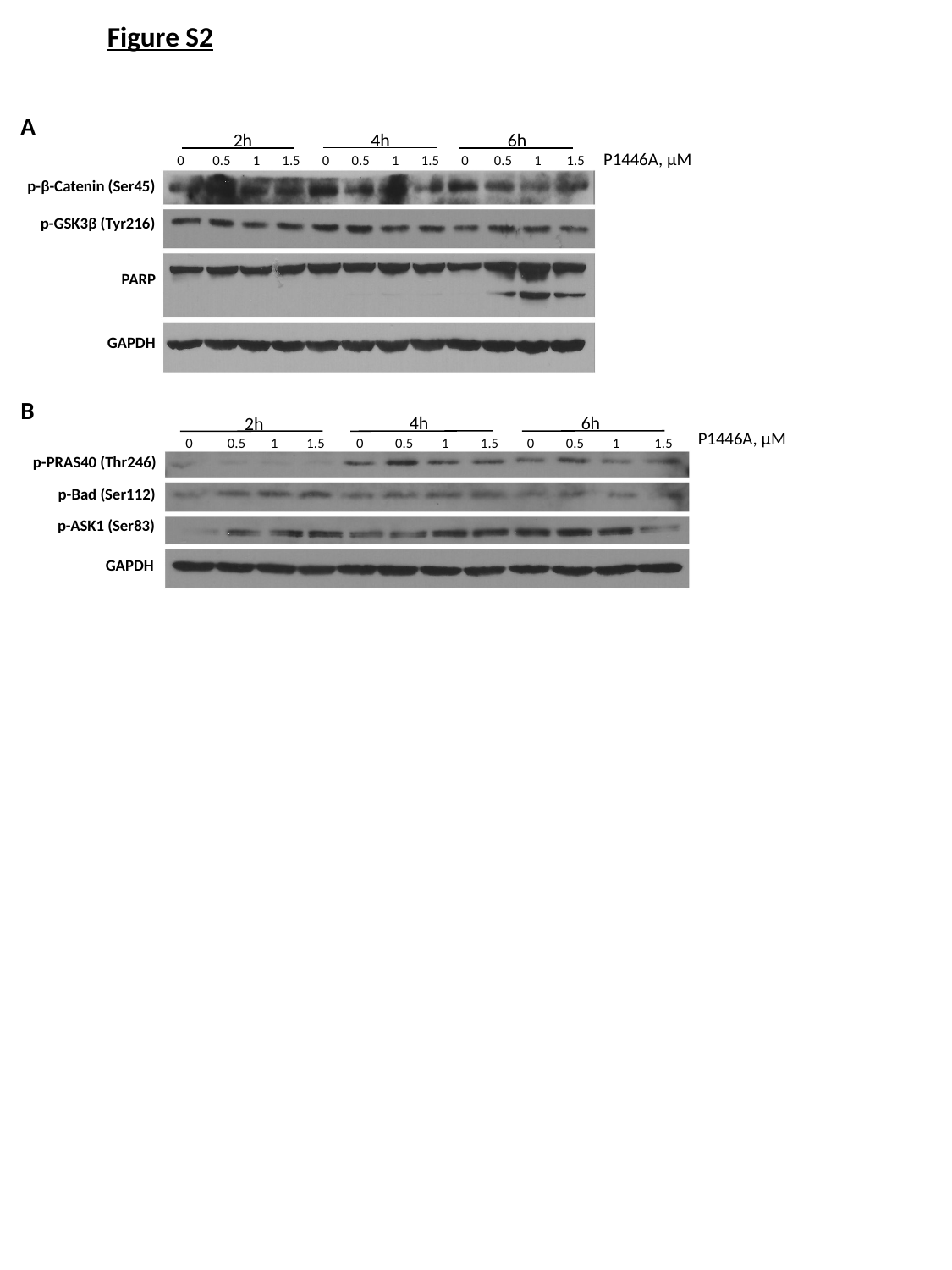

# Figure S2
A
4h
6h
2h
 P1446A, µM
 0 0.5 1 1.5 0 0.5 1 1.5 0 0.5 1 1.5
p-β-Catenin (Ser45)
p-GSK3β (Tyr216)
PARP
GAPDH
B
6h
4h
2h
 P1446A, µM
 0 0.5 1 1.5 0 0.5 1 1.5 0 0.5 1 1.5
p-PRAS40 (Thr246)
p-Bad (Ser112)
p-ASK1 (Ser83)
GAPDH
